# (*N*-Benzoyl-*N*′,*N*′-diphenyl­thio­ureato-κ^2^ *S*,*O*)(η^4^-cyclo­octa-1,5-diene)rhodium(I)

**DOI:** 10.1107/S1600536812029753

**Published:** 2012-07-10

**Authors:** Stefan Warsink, Andreas Roodt

**Affiliations:** a Department of Chemistry, University of the Free State, PO Box 339, Bloemfontein 9300, South Africa

## Abstract

The title complex, [Rh(C_20_H_15_N_2_OS)(C_8_H_12_)], exhibits an essentially square-planar coordination environment around the Rh^I^ atom, which bears a bidentate cyclo­octa­diene ligand as well as a monoanionic bidentate benzoyl­thio­ureate ligand. The Rh^I^ atom, the S- and O-donor atoms and the alkene centroids of the cyclo­octa­diene ligand do not deviate by more than 0.031 Å from their least mean-squares plane.

## Related literature

For rhodium complexes containing related monoanionic bidentate ligands, see: Trzeciak *et al.* (2004 ▶); Roodt *et al.* (2011 ▶); Crous *et al.* (2005 ▶); Guiseppe *et al.* (2011 ▶); Venter *et al.* (2009 ▶). For bidentate thio­ureato ligands, see: Sacht *et al.* (2000*a* ▶,*b* ▶); Kemp *et al.* (1997 ▶). For Rh^I^ complexes bearing cyclo­octa­diene and *S*,*O*-bidentate ligands, see: Grim *et al.* (1991 ▶); Hesp *et al.* (2007 ▶). For Rh^I^ complexes bearing a thio­urea ligand and cyclo­octa­diene, see: Kotze *et al.* (2010 ▶); Cauzzi *et al.* (1995 ▶). For tris­ubstituted thio­urea ligands, see: Hernandez *et al.* (2003 ▶); Arslan *et al.* (2003 ▶).
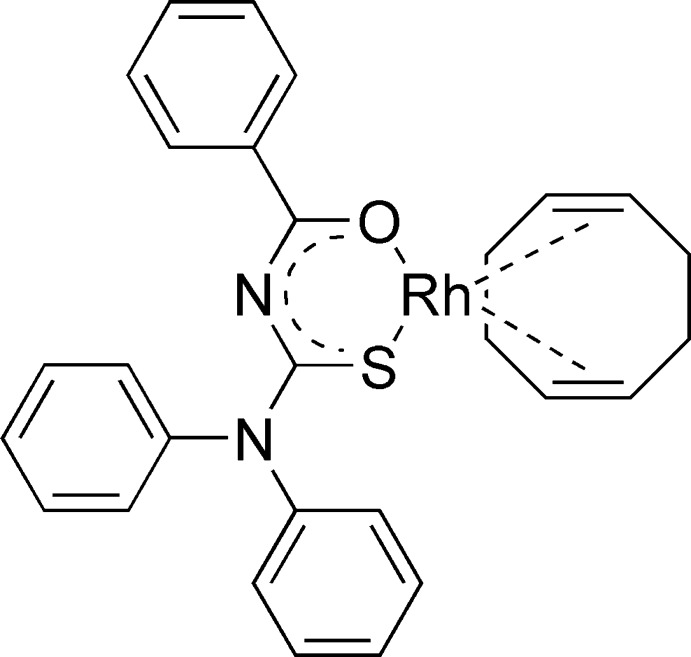


## Experimental

### 

#### Crystal data

[Rh(C_20_H_15_N_2_OS)(C_8_H_12_)]
*M* *_r_* = 542.50
Triclinic, 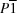

*a* = 9.8028 (4) Å
*b* = 11.2293 (5) Å
*c* = 11.5316 (5) Å
α = 90.408 (2)°
β = 91.684 (2)°
γ = 112.1831 (18)°
*V* = 1174.69 (9) Å^3^
*Z* = 2
Mo *K*α radiation
μ = 0.84 mm^−1^
*T* = 100 K
0.22 × 0.17 × 0.09 mm

#### Data collection

Bruker X8 APEXII 4K KappaCCD diffractometer
Absorption correction: multi-scan (*SADABS*; Bruker, 2007 ▶) *T*_min_ = 0.843, *T*_max_ = 0.927
12388 measured reflections
5583 independent reflections
5014 reflections with *I* > 2σ(*I*)
*R*_int_ = 0.025

#### Refinement

*R*[*F* ^2^ > 2σ(*F* ^2^)] = 0.028
*wR*(*F* ^2^) = 0.07
*S* = 1.04
5583 reflections
298 parameters
H-atom parameters constrained
Δρ_max_ = 0.66 e Å^−3^
Δρ_min_ = −1.01 e Å^−3^

### 

Data collection: *APEX2* (Bruker, 2007 ▶); cell refinement: *SAINT-Plus* (Bruker, 2007 ▶); data reduction: *SAINT-Plus*; program(s) used to solve structure: *SHELXS97* (Sheldrick, 2008 ▶); program(s) used to refine structure: *SHELXL97* (Sheldrick, 2008 ▶); molecular graphics: *Mercury* (Macrae *et al.*, 2008 ▶); software used to prepare material for publication: *WinGX* (Farrugia, 1999 ▶).

## Supplementary Material

Crystal structure: contains datablock(s) global, I. DOI: 10.1107/S1600536812029753/wm2654sup1.cif

Structure factors: contains datablock(s) I. DOI: 10.1107/S1600536812029753/wm2654Isup2.hkl

Additional supplementary materials:  crystallographic information; 3D view; checkCIF report

## Figures and Tables

**Table 1:** Selected bond lengths (Å)

Rh1—O5	2.0537 (16)
Rh1—C21	2.116 (2)
Rh1—C22	2.131 (2)
Rh1—C25	2.148 (2)
Rh1—C26	2.155 (2)
Rh1—S1	2.2942 (10)
C01—O5	1.263 (2)
C01—N1	1.330 (3)
C02—N1	1.346 (3)
C02—S1	1.726 (2)

**Fig. 1.**
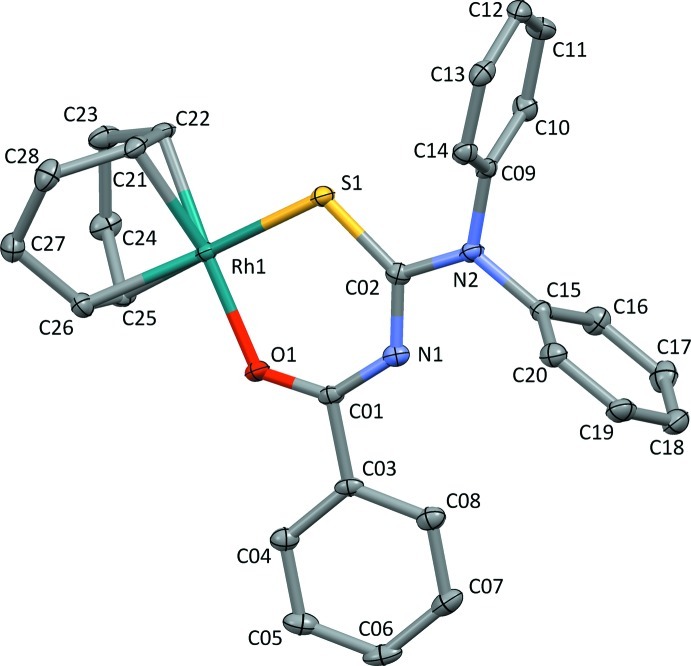
Molecular structure of (I). Displacement ellipsoids are drawn at the 50% probability level. H-atoms have been omitted for clarity.

## supplementary crystallographic information

### Comment

Rhodium complexes bearing bidentate ligands that bond through σ-interactions, such as β-diketonates and 8-hydroxyquinolates are well known (Trzeciak *et al.*, 2004; Guiseppe *et al.*, 2011). These bidentate ligands are compatible with a wide range of other ligands such as carbonyls and phosphines (Crous *et al.*, 2005; Venter *et al.*, 2009; Roodt *et al.*, 2011). Also regularly employed are thioureato ligands (Sacht *et al.*, 2000*a*,*b*; Kemp *et al.*, 1997).

The title compound [Rh(C_8_H_12_)(C_20_H_15_N_2_OS)], (I), bears a benzoyl-functionalized thioureato moiety (Arslan *et al.*, 2003), which can coordinate as a mono- or a bidentate ligand, depending on the metal and the other ligands present. With this specific ligand class, it was found that the peripheral substitution pattern significantly influences the coordination behaviour. When an *N*,*N'*,*N'*-trisubstituted thiourea ligand was employed, as is the case in this study, the thiourea coordinates as a monoanionic bidentate ligand, whereas an *N*,*N'*-disubstituted thiourea coordinates only through its sulfur-atom as a neutral monodentate ligand which is stabilized through intramolecular hydrogen bonding (Cauzzi *et al.*, 1995; Kotze *et al.*, 2010). One of these hydrogen bonds ensures that the sulfur and oxygen atoms are in a mutual *trans*-position, which stabilizes the pre-ligand in such a way that bidentate coordination is prevented. In the trisubstituted variation used in this study, this intramolecular interaction is not possible (Hernandez *et al.*, 2003), which enables the ligand to coordinate through its sulfur and oxygen atoms simultaneously. This structural report is only the third in which a rhodium complex bears both cyclooctadiene and *S*,*O*-bidentate ligands (Grim *et al.*, 1991; Hesp *et al.*, 2007).

The geometric parameters show that the rhodium(I) atom in the title compound has an essentially square planar coordination sphere. The deviation of the rhodium ion from the least mean squares plane, defined by the rhodium, oxygen and sulfur atoms and the centroids of the cyclooctadiene alkene bonds, is 0.001 Å. The donor atoms of the thioureato ligand and the centroids do not deviate more than 0.031 and 0.011 Å, respectively. The *S*,*O*-ligand exhibits a bite angle of 92.60 (5)°, and the cyclooctadiene ligand shows a bite angle of 87.90 (8)°. The bond lengths of the ligands to rhodium are all within the expected range for a compound of this type. The monoanionic ligand shows electron delocalization so that the bond lengths fall between those of single and double C—O, C—S and C—N bonds. There are no significant intermolecular interactions.

### Experimental

The title compound was prepared by adding 0.4 mmol of *N*-benzoyl-*N*,*N'*-diphenyl thiourea to a suspension of 0.2 mmol [RhCl(cod)]_2_ (cod is cyclooctadiene) in 5 ml of dichloromethane. The orange suspension changed into an orange solution, from which a yellow precipitate formed. After one hour of stirring, the yellow solid was isolated by filtration with a yield of 197 mg (90%, 0.36 mmol). ^1^H NMR (300 MHz, CDCl_3_): δ 7.66 (d, ^3^*J*(HH) = 7.4 Hz, 2H, *o*-benzoyl-H), 7.5–7.2 (m, 13H, 2x Ph, benzoyl), 4.71 (m, 2H, cod-alkene), 3.84 (m, 2H, cod-alkene), 2.6–2.4 (m, 4H, cod-alkane), 2.1–1.9 (m, 4H, cod-alkane). Yellow crystals of (I) were obtained by slow evaporation of a dichloromethane solution.

### Refinement

The hydrogen atoms were added geometrically and refined as riding on their parent atoms, with C—H distances of 0.95 Å for phenyl H atoms, of 1.00 Å for those bonded to *sp*^2^ C atoms and of 0.99 Å for those bonded to *sp*^2^ C atoms of the cyclooctadiene ligand. The thermal displacement coefficients *U*_iso_(H) were set to 1.2*U*_eq_(C) of the corresponding parent atoms.

### Crystal data

**Table:**

[Rh(C_20_H_15_N_2_OS)(C_8_H_12_)]	*Z* = 2
*M**_r_* = 542.50	*F*(000) = 556
Triclinic, *P*1	*D*_x_ = 1.534 Mg m^−^^3^
Hall symbol: -P 1	Mo *K*α radiation, λ = 0.71069 Å
*a* = 9.8028 (4) Å	Cell parameters from 7390 reflections
*b* = 11.2293 (5) Å	θ = 2.7–28.4°
*c* = 11.5316 (5) Å	µ = 0.84 mm^−^^1^
α = 90.408 (2)°	*T* = 100 K
β = 91.684 (2)°	Cuboid, yellow
γ = 112.1831 (18)°	0.22 × 0.17 × 0.09 mm
*V* = 1174.69 (9) Å^3^	

### Data collection

**Table:**

Bruker X8 APEXII 4K KappaCCD diffractometer	5583 independent reflections
Radiation source: fine-focus sealed tube	5014 reflections with *I* > 2σ(*I*)
Graphite monochromator	*R*_int_ = 0.025
ω and φ scans	θ_max_ = 28°, θ_min_ = 1.8°
Absorption correction: multi-scan (*SADABS*; Bruker, 2007)	*h* = −12→12
*T*_min_ = 0.843, *T*_max_ = 0.927	*k* = −14→14
12388 measured reflections	*l* = −14→15

### Refinement

**Table:**

Refinement on *F*^2^	Primary atom site location: structure-invariant direct methods
Least-squares matrix: full	Secondary atom site location: difference Fourier map
*R*[*F*^2^ > 2σ(*F*^2^)] = 0.028	Hydrogen site location: inferred from neighbouring sites
*wR*(*F*^2^) = 0.07	H-atom parameters constrained
*S* = 1.04	*w* = 1/[σ^2^(*F*_o_^2^) + (0.0309*P*)^2^ + 0.9591*P*] where *P* = (*F*_o_^2^ + 2*F*_c_^2^)/3
5583 reflections	(Δ/σ)_max_ = 0.001
298 parameters	Δρ_max_ = 0.66 e Å^−^^3^
0 restraints	Δρ_min_ = −1.01 e Å^−^^3^
0 constraints	

### Special details

**Table:**

Experimental. The intensity data was collected on a Bruker X8 ApexII 4 K Kappa CCD diffractometer using an exposure time of 10 s/frame. A total of 1166 frames was collected with a frame width of 0.5° covering up to θ = 28.00° with 98.3% completeness accomplished.
Geometry. All s.u.'s (except the s.u. in the dihedral angle between two l.s. planes) are estimated using the full covariance matrix. The cell s.u.'s are taken into account individually in the estimation of s.u.'s in distances, angles and torsion angles; correlations between s.u.'s in cell parameters are only used when they are defined by crystal symmetry. An approximate (isotropic) treatment of cell s.u.'s is used for estimating s.u.'s involving l.s. planes.
Refinement. Refinement of *F*^2^ against ALL reflections. The weighted *R*-factor *wR* and goodness of fit *S* are based on *F*^2^, conventional *R*-factors *R* are based on *F*, with *F* set to zero for negative *F*^2^. The threshold expression of *F*^2^ > 2σ(*F*^2^) is used only for calculating *R*-factors(gt) *etc*. and is not relevant to the choice of reflections for refinement. *R*-factors based on *F*^2^ are statistically about twice as large as those based on *F*, and *R*- factors based on ALL data will be even larger.

### Fractional atomic coordinates and isotropic or equivalent isotropic displacement parameters (Å^2^)

**Table:**

	*x*	*y*	*z*	*U*_iso_*/*U*_eq_	
Rh1	0.208984 (18)	0.353095 (14)	0.129434 (13)	0.01276 (6)	
C01	0.2830 (2)	0.59145 (18)	0.28171 (17)	0.0130 (4)	
C02	0.2300 (2)	0.41377 (19)	0.40937 (17)	0.0140 (4)	
C03	0.3203 (2)	0.73436 (19)	0.28531 (18)	0.0140 (4)	
C04	0.3589 (2)	0.8052 (2)	0.18426 (19)	0.0171 (4)	
H04	0.3621	0.7631	0.1132	0.02*	
C05	0.3927 (3)	0.9370 (2)	0.1869 (2)	0.0219 (5)	
H05	0.42	0.9849	0.118	0.026*	
C06	0.3866 (3)	0.9989 (2)	0.2904 (2)	0.0229 (5)	
H06	0.4089	1.0889	0.2921	0.027*	
C07	0.3477 (3)	0.9289 (2)	0.3912 (2)	0.0212 (5)	
H07	0.3431	0.9713	0.4617	0.025*	
C08	0.3154 (2)	0.7970 (2)	0.38982 (19)	0.0181 (4)	
H08	0.2901	0.7498	0.4593	0.022*	
C09	0.1983 (2)	0.26329 (18)	0.56941 (17)	0.0139 (4)	
C10	0.0552 (2)	0.1837 (2)	0.59464 (18)	0.0175 (4)	
H10	−0.0243	0.2112	0.5809	0.021*	
C11	0.0300 (3)	0.0630 (2)	0.64042 (19)	0.0206 (4)	
H11	−0.0675	0.007	0.6568	0.025*	
C12	0.1464 (3)	0.0243 (2)	0.66217 (18)	0.0191 (4)	
H12	0.1286	−0.0581	0.6934	0.023*	
C13	0.2891 (3)	0.1056 (2)	0.63844 (18)	0.0177 (4)	
H13	0.3688	0.0791	0.6546	0.021*	
C14	0.3161 (2)	0.22573 (19)	0.59104 (18)	0.0160 (4)	
H14	0.4135	0.2811	0.5738	0.019*	
C15	0.2466 (2)	0.49150 (19)	0.60976 (17)	0.0146 (4)	
C16	0.1290 (3)	0.5237 (2)	0.63976 (19)	0.0193 (4)	
H16	0.0346	0.4806	0.6035	0.023*	
C17	0.1497 (3)	0.6194 (2)	0.7232 (2)	0.0229 (5)	
H17	0.0696	0.6423	0.7437	0.028*	
C18	0.2883 (3)	0.6818 (2)	0.77667 (19)	0.0226 (5)	
H18	0.3028	0.7477	0.8332	0.027*	
C19	0.4047 (3)	0.6476 (2)	0.74738 (19)	0.0206 (5)	
H19	0.4988	0.6895	0.7846	0.025*	
C20	0.3844 (2)	0.5520 (2)	0.66356 (19)	0.0179 (4)	
H20	0.4642	0.5284	0.6434	0.021*	
C21	0.2178 (3)	0.17668 (19)	0.07235 (18)	0.0182 (4)	
H21	0.235	0.1239	0.1364	0.022*	
C22	0.0719 (2)	0.16845 (19)	0.06089 (18)	0.0170 (4)	
H22	0.0045	0.1112	0.1183	0.02*	
C23	−0.0034 (3)	0.1745 (2)	−0.0552 (2)	0.0214 (5)	
H23A	−0.109	0.1182	−0.0531	0.026*	
H23B	0.0411	0.1412	−0.1172	0.026*	
C24	0.0098 (3)	0.3117 (2)	−0.0848 (2)	0.0220 (5)	
H24A	0.0075	0.3199	−0.1701	0.026*	
H24B	−0.076	0.3266	−0.0546	0.026*	
C25	0.1499 (3)	0.4135 (2)	−0.03437 (18)	0.0178 (4)	
H25	0.1477	0.5018	−0.032	0.021*	
C26	0.2907 (3)	0.4124 (2)	−0.04074 (18)	0.0186 (4)	
H26	0.3709	0.4998	−0.0423	0.022*	
C27	0.3242 (3)	0.3089 (2)	−0.10575 (19)	0.0213 (5)	
H27A	0.4215	0.3481	−0.1412	0.026*	
H27B	0.249	0.2721	−0.169	0.026*	
C28	0.3250 (3)	0.2003 (2)	−0.0247 (2)	0.0228 (5)	
H28A	0.3	0.1199	−0.0712	0.027*	
H28B	0.4257	0.2227	0.0093	0.027*	
N1	0.2633 (2)	0.53861 (16)	0.38583 (15)	0.0151 (3)	
N2	0.2253 (2)	0.38996 (16)	0.52418 (15)	0.0144 (3)	
O5	0.27726 (18)	0.54244 (13)	0.18189 (12)	0.0178 (3)	
S1	0.19325 (6)	0.28310 (5)	0.31682 (4)	0.01539 (11)	

### Atomic displacement parameters (Å^2^)

**Table:**

	*U*^11^	*U*^22^	*U*^33^	*U*^12^	*U*^13^	*U*^23^
Rh1	0.01427 (9)	0.00972 (8)	0.01327 (9)	0.00346 (6)	−0.00045 (6)	−0.00109 (5)
C01	0.0090 (9)	0.0108 (9)	0.0177 (10)	0.0020 (7)	−0.0010 (7)	−0.0010 (7)
C02	0.0112 (10)	0.0144 (9)	0.0156 (10)	0.0042 (8)	−0.0005 (7)	0.0005 (7)
C03	0.0096 (9)	0.0101 (9)	0.0210 (10)	0.0025 (7)	−0.0025 (8)	−0.0006 (7)
C04	0.0155 (10)	0.0154 (10)	0.0194 (10)	0.0049 (8)	−0.0023 (8)	0.0003 (8)
C05	0.0198 (11)	0.0154 (10)	0.0285 (12)	0.0045 (9)	−0.0034 (9)	0.0055 (9)
C06	0.0176 (11)	0.0113 (9)	0.0398 (14)	0.0062 (9)	−0.0055 (10)	−0.0026 (9)
C07	0.0174 (11)	0.0167 (10)	0.0294 (12)	0.0070 (9)	−0.0037 (9)	−0.0087 (9)
C08	0.0153 (10)	0.0154 (10)	0.0225 (11)	0.0046 (8)	−0.0008 (8)	−0.0015 (8)
C09	0.0178 (10)	0.0105 (9)	0.0117 (9)	0.0037 (8)	−0.0011 (8)	−0.0017 (7)
C10	0.0146 (10)	0.0185 (10)	0.0194 (10)	0.0064 (9)	0.0012 (8)	0.0001 (8)
C11	0.0188 (11)	0.0147 (10)	0.0240 (11)	0.0013 (9)	0.0023 (9)	0.0010 (8)
C12	0.0278 (12)	0.0131 (9)	0.0157 (10)	0.0066 (9)	0.0030 (9)	0.0017 (8)
C13	0.0214 (11)	0.0173 (10)	0.0161 (10)	0.0096 (9)	−0.0031 (8)	−0.0031 (8)
C14	0.0146 (10)	0.0147 (10)	0.0169 (10)	0.0036 (8)	−0.0007 (8)	−0.0020 (8)
C15	0.0169 (10)	0.0122 (9)	0.0133 (9)	0.0038 (8)	0.0006 (8)	0.0020 (7)
C16	0.0172 (11)	0.0208 (11)	0.0198 (10)	0.0074 (9)	−0.0018 (8)	−0.0012 (8)
C17	0.0240 (12)	0.0252 (11)	0.0242 (11)	0.0144 (10)	0.0032 (9)	−0.0010 (9)
C18	0.0310 (13)	0.0164 (10)	0.0198 (11)	0.0085 (10)	0.0004 (9)	−0.0028 (8)
C19	0.0206 (11)	0.0144 (10)	0.0221 (11)	0.0015 (9)	−0.0025 (9)	−0.0022 (8)
C20	0.0179 (11)	0.0148 (10)	0.0207 (10)	0.0058 (8)	0.0005 (8)	0.0000 (8)
C21	0.0263 (12)	0.0120 (9)	0.0171 (10)	0.0084 (9)	−0.0016 (9)	−0.0026 (8)
C22	0.0198 (11)	0.0089 (9)	0.0196 (10)	0.0025 (8)	−0.0016 (8)	−0.0035 (7)
C23	0.0216 (12)	0.0159 (10)	0.0236 (11)	0.0040 (9)	−0.0057 (9)	−0.0037 (8)
C24	0.0233 (12)	0.0205 (11)	0.0226 (11)	0.0094 (9)	−0.0064 (9)	−0.0014 (9)
C25	0.0256 (12)	0.0125 (9)	0.0145 (10)	0.0068 (9)	−0.0045 (8)	0.0008 (7)
C26	0.0232 (12)	0.0158 (10)	0.0140 (10)	0.0040 (9)	0.0018 (8)	0.0014 (8)
C27	0.0249 (12)	0.0232 (11)	0.0169 (10)	0.0099 (10)	0.0043 (9)	−0.0005 (8)
C28	0.0254 (12)	0.0251 (11)	0.0218 (11)	0.0142 (10)	0.0001 (9)	−0.0044 (9)
N1	0.0148 (9)	0.0123 (8)	0.0174 (9)	0.0044 (7)	−0.0007 (7)	−0.0003 (6)
N2	0.0151 (9)	0.0104 (8)	0.0152 (8)	0.0023 (7)	−0.0012 (7)	−0.0016 (6)
O5	0.0244 (8)	0.0101 (7)	0.0153 (7)	0.0026 (6)	0.0004 (6)	−0.0016 (5)
S1	0.0199 (3)	0.0101 (2)	0.0143 (2)	0.0037 (2)	−0.00070 (19)	−0.00073 (18)

### Geometric parameters (Å, º)

**Table:**

Rh1—O5	2.0537 (16)	C14—H14	0.95
Rh1—C21	2.116 (2)	C15—C16	1.384 (3)
Rh1—C22	2.131 (2)	C15—C20	1.387 (3)
Rh1—C25	2.148 (2)	C15—N2	1.453 (3)
Rh1—C26	2.155 (2)	C16—C17	1.390 (3)
Rh1—S1	2.2942 (10)	C16—H16	0.95
C01—O5	1.263 (2)	C17—C18	1.393 (3)
C01—N1	1.330 (3)	C17—H17	0.95
C01—C03	1.505 (3)	C18—C19	1.384 (3)
C02—N1	1.346 (3)	C18—H18	0.95
C02—N2	1.351 (3)	C19—C20	1.393 (3)
C02—S1	1.726 (2)	C19—H19	0.95
C03—C04	1.395 (3)	C20—H20	0.95
C03—C08	1.403 (3)	C21—C22	1.400 (3)
C04—C05	1.389 (3)	C21—C28	1.514 (3)
C04—H04	0.95	C21—H21	1
C05—C06	1.390 (3)	C22—C23	1.524 (3)
C05—H05	0.95	C22—H22	1
C06—C07	1.389 (3)	C23—C24	1.538 (3)
C06—H06	0.95	C23—H23A	0.99
C07—C08	1.392 (3)	C23—H23B	0.99
C07—H07	0.95	C24—C25	1.512 (3)
C08—H08	0.95	C24—H24A	0.99
C09—C14	1.388 (3)	C24—H24B	0.99
C09—C10	1.390 (3)	C25—C26	1.389 (3)
C09—N2	1.449 (2)	C25—H25	1
C10—C11	1.394 (3)	C26—C27	1.520 (3)
C10—H10	0.95	C26—H26	1
C11—C12	1.382 (3)	C27—C28	1.544 (3)
C11—H11	0.95	C27—H27A	0.99
C12—C13	1.388 (3)	C27—H27B	0.99
C12—H12	0.95	C28—H28A	0.99
C13—C14	1.392 (3)	C28—H28B	0.99
C13—H13	0.95		
			
O5—Rh1—C21	160.27 (8)	C18—C17—H17	120.1
O5—Rh1—C22	160.44 (8)	C19—C18—C17	120.0 (2)
C21—Rh1—C22	38.48 (9)	C19—C18—H18	120
O5—Rh1—C25	86.38 (7)	C17—C18—H18	120
C21—Rh1—C25	98.03 (8)	C18—C19—C20	120.3 (2)
C22—Rh1—C25	81.92 (8)	C18—C19—H19	119.9
O5—Rh1—C26	89.94 (7)	C20—C19—H19	119.9
C21—Rh1—C26	82.25 (8)	C15—C20—C19	119.4 (2)
C22—Rh1—C26	90.31 (9)	C15—C20—H20	120.3
C25—Rh1—C26	37.66 (9)	C19—C20—H20	120.3
O5—Rh1—S1	92.60 (5)	C22—C21—C28	125.9 (2)
C21—Rh1—S1	89.41 (6)	C22—C21—Rh1	71.31 (12)
C22—Rh1—S1	93.30 (6)	C28—C21—Rh1	109.86 (14)
C25—Rh1—S1	160.68 (7)	C22—C21—H21	113.9
C26—Rh1—S1	161.63 (7)	C28—C21—H21	113.9
O5—C01—N1	131.01 (18)	Rh1—C21—H21	113.9
O5—C01—C03	115.41 (17)	C21—C22—C23	123.5 (2)
N1—C01—C03	113.57 (18)	C21—C22—Rh1	70.21 (12)
N1—C02—N2	113.29 (18)	C23—C22—Rh1	113.14 (14)
N1—C02—S1	130.18 (16)	C21—C22—H22	114.1
N2—C02—S1	116.53 (15)	C23—C22—H22	114.1
C04—C03—C08	119.53 (19)	Rh1—C22—H22	114.1
C04—C03—C01	120.11 (19)	C22—C23—C24	112.68 (18)
C08—C03—C01	120.36 (18)	C22—C23—H23A	109.1
C05—C04—C03	120.4 (2)	C24—C23—H23A	109.1
C05—C04—H04	119.8	C22—C23—H23B	109.1
C03—C04—H04	119.8	C24—C23—H23B	109.1
C04—C05—C06	120.1 (2)	H23A—C23—H23B	107.8
C04—C05—H05	120	C25—C24—C23	112.62 (18)
C06—C05—H05	120	C25—C24—H24A	109.1
C07—C06—C05	119.9 (2)	C23—C24—H24A	109.1
C07—C06—H06	120.1	C25—C24—H24B	109.1
C05—C06—H06	120.1	C23—C24—H24B	109.1
C06—C07—C08	120.5 (2)	H24A—C24—H24B	107.8
C06—C07—H07	119.7	C26—C25—C24	125.8 (2)
C08—C07—H07	119.7	C26—C25—Rh1	71.44 (12)
C07—C08—C03	119.6 (2)	C24—C25—Rh1	110.18 (14)
C07—C08—H08	120.2	C26—C25—H25	113.8
C03—C08—H08	120.2	C24—C25—H25	113.8
C14—C09—C10	121.27 (19)	Rh1—C25—H25	113.8
C14—C09—N2	119.49 (19)	C25—C26—C27	123.3 (2)
C10—C09—N2	119.20 (19)	C25—C26—Rh1	70.90 (12)
C09—C10—C11	119.0 (2)	C27—C26—Rh1	112.49 (14)
C09—C10—H10	120.5	C25—C26—H26	114.2
C11—C10—H10	120.5	C27—C26—H26	114.2
C12—C11—C10	120.3 (2)	Rh1—C26—H26	114.2
C12—C11—H11	119.9	C26—C27—C28	111.68 (18)
C10—C11—H11	119.9	C26—C27—H27A	109.3
C11—C12—C13	120.1 (2)	C28—C27—H27A	109.3
C11—C12—H12	119.9	C26—C27—H27B	109.3
C13—C12—H12	119.9	C28—C27—H27B	109.3
C12—C13—C14	120.4 (2)	H27A—C27—H27B	107.9
C12—C13—H13	119.8	C21—C28—C27	113.09 (19)
C14—C13—H13	119.8	C21—C28—H28A	109
C09—C14—C13	118.9 (2)	C27—C28—H28A	109
C09—C14—H14	120.6	C21—C28—H28B	109
C13—C14—H14	120.6	C27—C28—H28B	109
C16—C15—C20	120.8 (2)	H28A—C28—H28B	107.8
C16—C15—N2	120.08 (19)	C01—N1—C02	126.80 (18)
C20—C15—N2	119.10 (19)	C02—N2—C09	122.68 (17)
C15—C16—C17	119.7 (2)	C02—N2—C15	121.22 (17)
C15—C16—H16	120.1	C09—N2—C15	116.10 (16)
C17—C16—H16	120.1	C01—O5—Rh1	130.27 (13)
C16—C17—C18	119.9 (2)	C02—S1—Rh1	108.49 (7)
C16—C17—H17	120.1		
			
O5—C01—C03—C04	−5.8 (3)	C21—Rh1—C25—C26	−66.06 (14)
N1—C01—C03—C04	173.41 (18)	C22—Rh1—C25—C26	−101.12 (14)
O5—C01—C03—C08	173.64 (19)	S1—Rh1—C25—C26	−177.86 (13)
N1—C01—C03—C08	−7.2 (3)	O5—Rh1—C25—C24	−143.13 (16)
C08—C03—C04—C05	0.2 (3)	C21—Rh1—C25—C24	56.21 (17)
C01—C03—C04—C05	179.6 (2)	C22—Rh1—C25—C24	21.15 (16)
C03—C04—C05—C06	−0.7 (3)	C26—Rh1—C25—C24	122.3 (2)
C04—C05—C06—C07	0.5 (3)	S1—Rh1—C25—C24	−55.6 (3)
C05—C06—C07—C08	0.3 (3)	C24—C25—C26—C27	3.0 (3)
C06—C07—C08—C03	−0.8 (3)	Rh1—C25—C26—C27	104.9 (2)
C04—C03—C08—C07	0.6 (3)	C24—C25—C26—Rh1	−101.9 (2)
C01—C03—C08—C07	−178.84 (19)	O5—Rh1—C26—C25	−84.15 (13)
C14—C09—C10—C11	−1.3 (3)	C21—Rh1—C26—C25	114.02 (13)
N2—C09—C10—C11	−178.66 (19)	C22—Rh1—C26—C25	76.28 (13)
C09—C10—C11—C12	1.1 (3)	S1—Rh1—C26—C25	177.75 (14)
C10—C11—C12—C13	0.0 (3)	O5—Rh1—C26—C27	156.78 (16)
C11—C12—C13—C14	−1.0 (3)	C21—Rh1—C26—C27	−5.04 (16)
C10—C09—C14—C13	0.3 (3)	C22—Rh1—C26—C27	−42.78 (17)
N2—C09—C14—C13	177.69 (18)	C25—Rh1—C26—C27	−119.1 (2)
C12—C13—C14—C09	0.8 (3)	S1—Rh1—C26—C27	58.7 (3)
C20—C15—C16—C17	1.2 (3)	C25—C26—C27—C28	−94.1 (3)
N2—C15—C16—C17	178.88 (19)	Rh1—C26—C27—C28	−12.9 (2)
C15—C16—C17—C18	−0.4 (3)	C22—C21—C28—C27	44.9 (3)
C16—C17—C18—C19	−0.6 (3)	Rh1—C21—C28—C27	−35.9 (2)
C17—C18—C19—C20	0.7 (3)	C26—C27—C28—C21	32.2 (3)
C16—C15—C20—C19	−1.0 (3)	O5—C01—N1—C02	−0.6 (4)
N2—C15—C20—C19	−178.70 (18)	C03—C01—N1—C02	−179.59 (19)
C18—C19—C20—C15	0.0 (3)	N2—C02—N1—C01	176.38 (19)
O5—Rh1—C21—C22	−167.87 (17)	S1—C02—N1—C01	−3.7 (3)
C25—Rh1—C21—C22	−66.06 (14)	N1—C02—N2—C09	−177.07 (18)
C26—Rh1—C21—C22	−100.37 (14)	S1—C02—N2—C09	3.0 (3)
S1—Rh1—C21—C22	96.05 (12)	N1—C02—N2—C15	3.3 (3)
O5—Rh1—C21—C28	−45.4 (3)	S1—C02—N2—C15	−176.63 (15)
C22—Rh1—C21—C28	122.4 (2)	C14—C09—N2—C02	88.4 (2)
C25—Rh1—C21—C28	56.38 (17)	C10—C09—N2—C02	−94.2 (2)
C26—Rh1—C21—C28	22.07 (15)	C14—C09—N2—C15	−92.0 (2)
S1—Rh1—C21—C28	−141.51 (15)	C10—C09—N2—C15	85.4 (2)
C28—C21—C22—C23	3.8 (3)	C16—C15—N2—C02	84.2 (3)
Rh1—C21—C22—C23	105.22 (19)	C20—C15—N2—C02	−98.1 (2)
C28—C21—C22—Rh1	−101.4 (2)	C16—C15—N2—C09	−95.4 (2)
O5—Rh1—C22—C21	167.77 (18)	C20—C15—N2—C09	82.3 (2)
C25—Rh1—C22—C21	113.92 (14)	N1—C01—O5—Rh1	8.8 (3)
C26—Rh1—C22—C21	77.09 (13)	C03—C01—O5—Rh1	−172.15 (13)
S1—Rh1—C22—C21	−84.89 (12)	C21—Rh1—O5—C01	−104.8 (3)
O5—Rh1—C22—C23	48.8 (3)	C22—Rh1—O5—C01	98.2 (3)
C21—Rh1—C22—C23	−119.0 (2)	C25—Rh1—O5—C01	151.38 (19)
C25—Rh1—C22—C23	−5.05 (17)	C26—Rh1—O5—C01	−171.10 (19)
C26—Rh1—C22—C23	−41.89 (17)	S1—Rh1—O5—C01	−9.30 (18)
S1—Rh1—C22—C23	156.14 (16)	N1—C02—S1—Rh1	0.0 (2)
C21—C22—C23—C24	−92.8 (3)	N2—C02—S1—Rh1	179.91 (14)
Rh1—C22—C23—C24	−12.0 (3)	O5—Rh1—S1—C02	4.43 (9)
C22—C23—C24—C25	30.3 (3)	C21—Rh1—S1—C02	164.79 (10)
C23—C24—C25—C26	47.4 (3)	C22—Rh1—S1—C02	−156.91 (10)
C23—C24—C25—Rh1	−33.8 (2)	C25—Rh1—S1—C02	−82.05 (19)
O5—Rh1—C25—C26	94.60 (13)	C26—Rh1—S1—C02	102.1 (2)

## References

[bb1] Arslan, H., Flörke, U. & Külcü, N. (2003). *Acta Cryst.* E**59**, o641–o642.

[bb2] Bruker (2007). *APEX2*, *SAINT-Plus* and *SADABS* Bruker AXS Inc., Madison, Wisconsin, USA.

[bb3] Cauzzi, D., Lanfranchi, M., Marzolini, G., Predieri, G., Tiripicchio, A., Costa, M. & Zanoni, R. (1995). *J. Organomet. Chem.* **488**, 115–125.

[bb4] Crous, R., Datt, M., Foster, D., Bennie, L., Steenkamp, C., Huyser, J., Kirsten, L., Steyl, G. & Roodt, A. (2005). *Dalton Trans.* pp. 1108–1116. 10.1039/b416917d 15739014

[bb5] Farrugia, L. J. (1999). *J. Appl. Cryst.* **32**, 837–838.

[bb6] Grim, S. O., Kettler, P. B. & Thoden, J. B. (1991). *Organometallics*, **10**, 2399–2403.

[bb7] Guiseppe, A. D., Casterlenas, R., Perez-Torrente, J. J., Lahoz, F. H., Polo, V. & Oro, L. A. (2011). *Angew. Chem. Int. Ed.* **50**, 3938–3942. 10.1002/anie.201007238 21442694

[bb8] Hernandez, W., Spodine, E., Munoz, J. C., Beyer, L., Schroder, U., Ferreira, J. & Pavani, M. (2003). *Bioinorg. Chem. Appl.* **1**, 271–284. 10.1155/S1565363303000219 PMC2267062 18365059

[bb9] Hesp, K. D., Wechsler, D., Cipot, J., Myers, A., McDonald, R., Ferguson, M. J., Schatte, G. & Stradiotto, M. (2007). *Organometallics*, **26**, 5430–5437.

[bb10] Kemp, G., Purcell, W., Roodt, A. & Koch, K. R. (1997). *J. Chem. Soc. Dalton Trans.* pp. 4481–4483.

[bb11] Kotze, P. D. R., Roodt, A., Venter, J. A. & Otto, S. (2010). *Acta Cryst.* E**66**, m1028–m1029. 10.1107/S1600536810029740 PMC3007502 21588103

[bb12] Macrae, C. F., Bruno, I. J., Chisholm, J. A., Edgington, P. R., McCabe, P., Pidcock, E., Rodriguez-Monge, L., Taylor, R., van de Streek, J. & Wood, P. A. (2008). *J. Appl. Cryst.* **41**, 466–470.

[bb13] Roodt, A., Visser, H. G. & Brink, A. (2011). *Crystallogr. Rev.* **17**, 241–280.

[bb14] Sacht, C. I., Datt, M. S., Otto, S. & Roodt, A. (2000*a*). *J. Chem. Soc. Dalton Trans.* pp. 727–73.

[bb15] Sacht, C. I., Datt, M. S., Otto, S. & Roodt, A. (2000*b*). *J. Chem. Soc. Dalton Trans.* pp. 4579–4586.

[bb16] Sheldrick, G. M. (2008). *Acta Cryst.* A**64**, 112–122. 10.1107/S0108767307043930 18156677

[bb17] Trzeciak, A. M., Borak, B., Cinnik, Z., Ziolkowski, J. J., Guedes da Silva, M. F. C. & Pombeiro, A. J. L. (2004). *Eur. J. Inorg. Chem.* pp. 1411–1419.

[bb18] Venter, J. A., Purcell, W., Visser, H. G. & Muller, T. J. (2009). *Acta Cryst.* E**65**, m1578. 10.1107/S1600536809047321 PMC2972147 21578610

